# A Lightweight Accountable Parallel Blockchain Architecture Based on Redactable Blockchain for Agri-Food Traceability

**DOI:** 10.3390/foods14040623

**Published:** 2025-02-13

**Authors:** Feng Chen, Chunjiang Zhao, Xinting Yang, Na Luo, Chuanheng Sun

**Affiliations:** 1 College of Information and Electrical Engineering, Shenyang Agricultural University, Shenyang 110866, China; chenf@stu.syau.edu.cn (F.C.); zhaocj@nercita.org.cn (C.Z.); 2 National Engineering Research Center for Information Technology in Agriculture, Beijing 100097, China; yangxt@nercita.org.cn (X.Y.); luon@nercita.org.cn (N.L.); 3 National Engineering Research Center for Quality and Safety Traceability Technology and Application of Agricultural Products, Beijing 100097, China

**Keywords:** agri-food supply chain, redactable blockchain, recoverability

## Abstract

Agri-food safety issues have received widespread attention globally. The emergence of blockchain technology (BCT) effectively addresses trust issues in the agri-food supply chain traceability system (AFSCTS). However, the append-only feature of blockchain has led to continuous linear data growth in BCT-based AFSCTSs, which increases the equipment requirements and has become a bottleneck for BCT-based AFSCTS applications. The storage capacity required by BCT-based AFSCTSs can be effectively reduced by deleting expired data, thereby reducing the storage pressure on blockchain devices and lowering the device requirements. In this paper, we propose an AFSCTS architecture that incorporates redactable blockchain and InterPlanetary file system (IPFS) technologies to achieve traceability with low storage pressure, using the wheat supply chain as a proof of concept. Firstly, the key links were analyzed in agri-food traceability and the demand was proposed for agri-food blockchain traceability based on the timeliness of traceability data. Secondly, a lightweight accountable parallel blockchain architecture called LAP-chain is proposed. This architecture utilizes redactable blockchain technology to offload expired agri-food traceability data to IPFS, thereby reducing the storage pressure on blockchain devices and ensuring data accountability through IPFS. Finally, we evaluate the correctness, collision resistance, and storage performance of the LAP-chain built on the Ethereum private chain. The results show that when expired agri-food traceability data are permanently retained, the storage capacity of the proposed architecture is only 52.38% of that of the traditional blockchain traceability architecture, after running continuously for 36 months. When traceability data of expired agri-food are deleted in accordance with the food laws and regulations of various countries, the storage capacity of the proposed architecture can be reduced from a linear level to a constant level compared to the traditional blockchain traceability architecture. The proposed architecture has the potential to contribute to improving the safety and quality of agri-food.

## 1. Introduction

Agri-food safety issues have received widespread global attention, and countries around the world have begun to focus on traceability in the agri-food supply chain. The EU General Food Law, promulgated in 2002, requires the agri-food sector to establish a comprehensive agri-food supply chain traceability system (AFSCTS) to enable timely and accurate recalls and transparently convey information to consumers [1]. Similarly, the Food Safety Law of the People’s Republic of China mandates that food producers establish a food safety traceability system to ensure food traceability [2]. However, the agri-food vertical domain, spanning from farm to fork, has features such as long timeframes, extensive spatial coverage, and involvement of many companies. These characteristics can lead to issues such as a discontinuous tracking chain and unauthorized modifications to traceability information [3]. As a result, consumers have become distrustful of agri-food supply chain (AFSC) traceability information accessed through the internet. Ensuring the security and trustworthiness of agri-food traceability data in a complex and dynamic environment remains a significant challenge. Fortunately, the emergence of blockchain technology (BCT) effectively addresses the trust issues in AFSCTSs [4]. BCT possesses key characteristics such as decentralization, immutability, transparency, and auditability [5]. BCT-based AFSCTSs can effectively resolve trust issues in data transmission between upstream and downstream enterprises in the supply chain. They also provide technical support to address problems such as information chain discontinuity and opacity caused by the long traceability chain, decentralized production, and heterogeneous information sources in traditional traceability systems [6].

However, the append-only and tamper-proof nature of BCT has also introduced certain challenges. Blockchain is an ever-growing, permanent record of data blocks. As of September 2024, the storage capacity of Ethereum archive nodes using the Geth version has exceeded 19 TB [7]. AFSC traceability involves numerous stages and generates vast amounts of data. The data storage overhead of blockchain and the associated hardware costs constitute one of the primary additional expenses incurred by adopting BCT [8,9]. This consequently results in low user willingness to adopt BCT-based AFSCTSs. Consequently, the continuous growth of AFSC traceability data increases the performance requirements for devices in a BCT-based AFSCTS, which has become a primary bottleneck for the large-scale application of BCT-based AFSCTSs [10]. Due to the massive volume of AFSC traceability data, blockchain devices will eventually run out of storage space to accommodate the entire chain. Thereby, a primary challenge for BCT-based AFSCTSs is scalability in storage capacity. Considering that agri-food has a shelf life, AFSC traceability data are time-sensitive. The query frequency for expired agri-food traceability data is extremely low, and its value is limited. The storage requirements of the blockchain-based agri-food supply chain can be effectively reduced by deleting expired data, thus alleviating storage pressure on blockchain devices and lowering device requirements. Additionally, even if food safety issues arise for agri-food products beyond their shelf life, such products should still be traceable to meet traceability demands.

Consequently, our motivation is to establish a lightweight accountable parallel blockchain architecture. The main contributions of this work are as follows.

(1) This paper analyzes the key links of agri-food traceability and analyzes the timeliness of the traceability data of each link. Based on the timeliness of agri-food traceability data, the demand for agri-food blockchain traceability is proposed.
(2) We propose a lightweight accountable parallel blockchain architecture, called LAP-chain, which is proposed to reduce the storage pressure of the blockchain equipment. In addition, the architecture ensures the accountability of data by offloading expired data to the InterPlanetary file system.
(3) LAP-chain was built using Ethereum private chain, and the correctness, collision resistance, and storage performance of the architecture were analyzed. The results show that the proposed architecture is capable of significantly reducing storage capacity while ensuring traceability.

This paper proposes incorporating an agri-food traceability system and a redactable blockchain to achieve traceability with low storage pressure, taking the wheat supply chain as proof of concept. The remainder of this paper is organized as follows. Section 2 reviews related works on agri-food traceability systems and redactable blockchains. Section 3 analyzes the key links of agri-food traceability and presents the proposed agri-food redactable blockchain traceability model. Section 4 introduces the proposed key technologies of redactable blockchain. In Section 5, the integration of redactable blockchain and agri-food blockchain traceability in the wheat supply chain experiment is reported. Finally, Section 6 concludes the paper.

## 2. Related Works

The International Organization for Standardization defines traceability in the agri-food sector as “the ability to track the movement of food through designated stages of production, processing, and distribution” [11]. Traditional AFSCTSs face trust issues. BCT-based AFSCTSs effectively addresses these trust challenges and provides technical support to alleviate issues related to information chain disruptions and lack of transparency, which are caused by long traceability chains, decentralized production, and heterogeneous information sources in conventional traceability systems. Consequently, BCT-based AFSCTSs are widely applied in the AFSC field. A BCT-based AFSCTS with a multi-chain architecture was proposed for the Portuguese hams’ production scenario, which can guarantee the immutability, reliability, and transparency of the data along the value chain [12]. A blockchain-based traceability framework proposed for agri-food supply chains, focusing on honey and coriander powder, emphasizes blockchain’s potential to enhance transparency, data integrity, and operational efficiency in high-value food supply chains [13]. An AFSC traceability framework that integrates BCT with digital tools such as QR codes, Radio Frequency Identification, and Near-Field Communication has been proposed to ensure traceability, transparency, and efficiency across the entire network from farmers to consumers, with a specific focus on the Vietnamese cashew nut business [14]. A BCT-based AFSC traceability model is proposed to balance agricultural expert knowledge, value chain planning, and digital technology, aiming to provide tamper-proof, transparent, and secure traceability specifically for the industrial hemp production sector [15]. A BCT-based AFSCTS is proposed to track food production and distribution from farm to retail, enabling consumers to access information about food origins and production by scanning a QR code on the product. This system aims to enhance effectiveness, transparency, and sustainability through accurate product traceability [16]. Although BCT-based AFSCTSs are widely utilized in the AFSC field, they also have several significant drawbacks. The continuous linear growth of agri-food supply chain blockchain data has raised the threshold of blockchain devices and become the bottleneck of BCT-based AFSCTSs large-scale applications.

To address the issue of high storage pressure in BCT, various solutions have been proposed, including the on-chain and off-chain data storage method [17,18], BCT and IPFS storage structure [19,20], sharding [21,22], Holochain [23], RS erasure code [24], and redactable blockchain [25]. Among these, redactable blockchain has garnered significant attention for its ability to modify or delete specific data without compromising the integrity of the blockchain. A hard fork, the most common method for editing blockchains, allows modifications to historical data on the blockchain [26]. However, a hard fork does not completely delete historical data from the blockchain, as the pre-modification data remain preserved. Therefore, a hard fork cannot effectively reduce blockchain storage requirements. To address the issue of improper data stored in transaction outputs within the local storage of nodes, a functionality-preserving local erasure technique has been proposed [27]. This approach modifies the ScriptPubKey field of transaction outputs while securely storing the original information in an erasure database prior to modification. Functionality-preserving local erasure is classified as a form of logical redaction, wherein data are altered locally without affecting the integrity of the original blockchain data. Consequently, this method ensures that the storage capacity of the blockchain remains unaffected while enabling the removal of improper data at the node level. An efficient redactable blockchain solution has been proposed for the permissionless setting, offering seamless integration with Bitcoin [28]. However, this approach lacks applicability to a broader range of mainstream blockchain platforms. A redactable blockchain in a decentralized setting was proposed to address the issues of blockchain storage abuse and compliance with legal obligations [29]. An Identity management and authentication scheme was proposed based on redactable Blockchain for mobile networks, which employs chameleon hash functions to delete illegal user information from the blockchain while preserving the integrity of the block header [30]. A scheme based on redactable blockchain for managing shared healthcare data has been proposed, allowing users to encrypt data to safeguard privacy and decrypt it when sharing medical information [31]. A redactable blockchain-based data management scheme for agri-food traceability introduces the chameleon hash function to facilitate data modification capabilities [32]. These studies leverage redactable blockchains to enable data modification or deletion, thereby reducing the storage burden of BCT-based AFSCTSs and ensuring compliance with legal obligations.

However, no studies have considered the time sensitivity of agri-food traceability data and the demand for agri-food traceability for agri-food outside of its shelf life in an agri-food blockchain traceability system. Consequently, there is an urgent need to propose a novel blockchain traceability system tailored for agri-food applications. Such a system should integrate a recoverable agri-food traceability mechanism within a redactable blockchain framework, enhancing reliability in terms of traceability while further reducing the storage capacity requirements of the blockchain. This is particularly important as certain types of transactions may lose their value over time, necessitating efficient data management strategies.

## 3. Parallel Blockchain Architecture for Agri-Food Traceability

### 3.1. Key Links of Agri-Food Supply Chain Traceability

**Figure 1:**
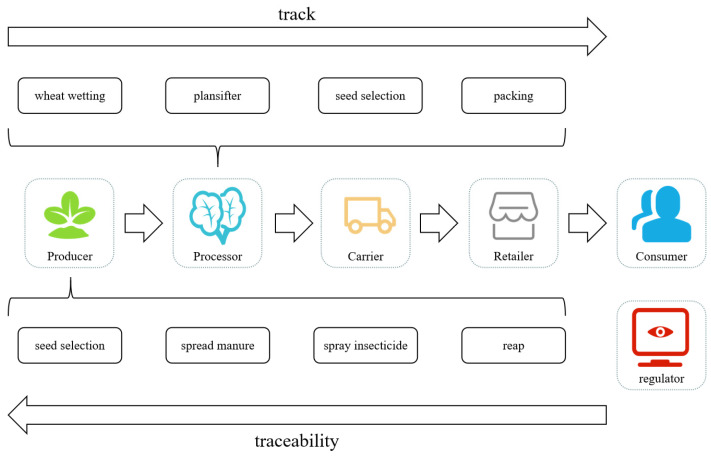
Key links of agri-food traceability.

An effective agri-food supply chain traceability system must collect the relevant data from stakeholders and define information owners at every stage of the agri-food supply chain. Then, the information must be clearly and effectively managed and presented to all stakeholders involved (e.g., customers, producers, retailers, carriers, and governmental agencies). The agri-food supply chain is characterized by numerous links, independent manufacturers, and a large amount of data to be recorded and transmitted. According to the characteristics of agri-food traceability information, the agri-food supply chain is divided into four stages from the perspective of traceability: the producer stage, the processing stage, the carrier stage, and the retail stage, before it finally enters the hands of end consumers, as shown in Figure 1. Each major link of different agri-foods contains different specific links. This study will use wheat flour as an example. The producer stage includes seed selection, spreading manure, spraying insecticide, reaping, and so on. Processors are responsible for wheat wetting, milling, plansifter packing, and so on.

The business information of each link in the agri-food supply chain is divided into source tracing information and general product information. Tracing the batch number information can realize the mutual correspondence of one code and one object. Consumers can find the corresponding product information by tracing the source code. Product information includes manufacturer information and qualification certificate information. Taking the processing stage as an example, the processing manufacturer records the processing link information of agri-food and traces the business information of the processing stage through the processing source code. In addition, the factory name, product name, manufacturing date, shelf life, and expiry date are recorded during the processing stage. The business information analysis of the agri-food supply chain can provide a basis for the subsequent establishment of the traceability model of the agri-food supply chain. The timeliness of supply chain information is shown in Table 1.

### 3.2. Design of Lightweight Accountable Parallel Blockchain Architecture

**Figure 2:**
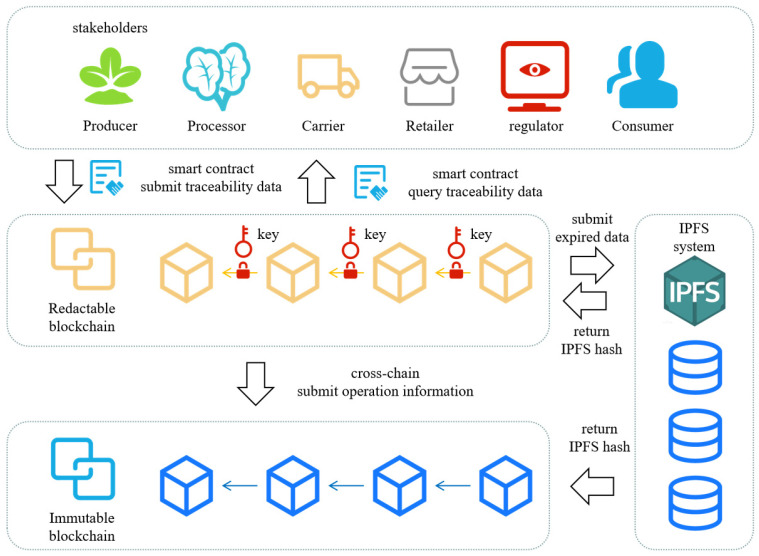
Lightweight accountable parallel blockchain architecture.

This paper provides a novel lightweight accountable parallel blockchain architecture, called LAP-chain, which realizes the offloading of time-effective agri-food traceability data while ensuring the accountability and trustworthiness of the agri-food traceability data. In the architecture, there are three entities involved: redactable blockchain, immutable blockchain, and InterPlanetary file system (IPFS), as shown in Figure 2. A detailed description is provided below.

(1) Redactable blockchain: redactable blockchain is used to store unexpired agri-food traceability data. The design details are in Section 4.
(2) Immutable blockchain: immutable blockchain is used to store the operation record of the redactable blockchain and IPFS hash of the agri-food traceability data after offload.
(3) IPFS: IPFS is used to store expired agri-food traceability data.

## 4. Design of Redactable Blockchain

### 4.1. Redactable Blockchain Design

**Figure 3:**
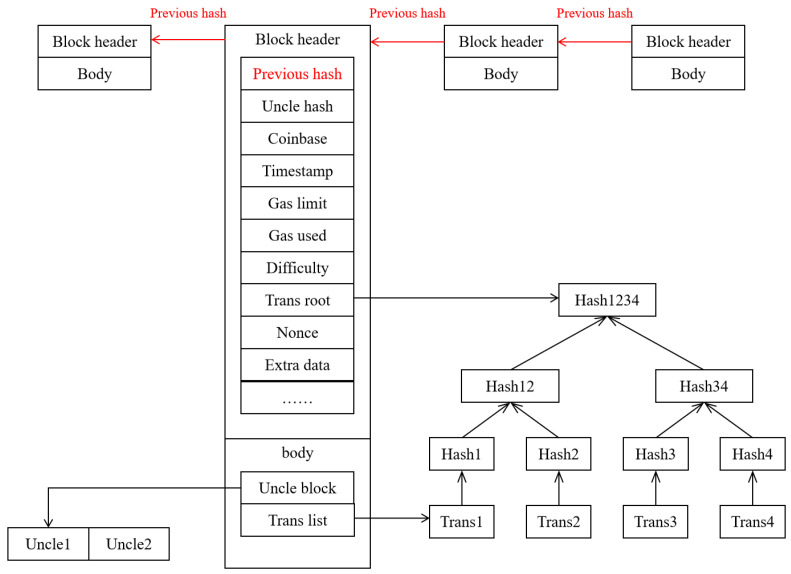
Blockchain data structure.

The key features of blockchain are decentralization and a tamper-proof and immutable blockchain data structure. The cascading effect of the hash chain, as shown in Figure 3, ensures that if anyone modifies the data in the blockchain, the “previous hash” pointer will be defective. It is difficult to recalculate the hash value in the next block. Therefore, the immutable advantage is that once the data are uploaded to the blockchain, as long as the blockchain continues to exist, the auditability and integrity will remain secure. However, the traditional blockchain architecture has fewer resources in terms of capacity. With the growth of the blockchain ledger scale, the cost of verifying and maintaining transactions on the ledger increases the waste of storage resources. Therefore, deleting expired data is crucial for the effective management of storage in the blockchain. However, due to the immutability of the blockchain, expired data cannot be deleted.

The chameleon hash function is proposed by Hugo Krawczyk [33]. It is a special one-way trapdoor function. Each chameleon hash has a corresponding trapdoor. Everyone who has the trapdoor can efficiently find the collisions of the corresponding hash function, but it is hard for one who does not have the trapdoor to find a collision of the corresponding hash function.

The previous hash in the block header on the first chain uses the chameleon hash function, which is composed of three parts:

Cham_hash = (KeyGen, Hash, Forge)(1)

Inside, KeyGen (*λ*) is used to calculate the trap gate and hash key through the input security parameters and it is recorded as follows:

KeyGen (*λ*) → (*TK*, *HK*) (2)

Hash (*HK*, *m*, *r*) is used to calculate the hash value through the hash key, message, and random number, and it is recorded as follows:

Hash (*HK*, *m*, *r*) → *y* (3)

Forge (*TK*, *m*, *r*, *m*′): Through the trap gate, message, random number, and message, another random number is calculated to satisfy Equation (4):

Hash (*HK*, *m*, *r*) = Hash(*HK*, *m*′, *r*′) (4)

Record as

Forge (*TK*, *m*, *r*, *m*′) → *r*′ (5)

**Figure 4:**
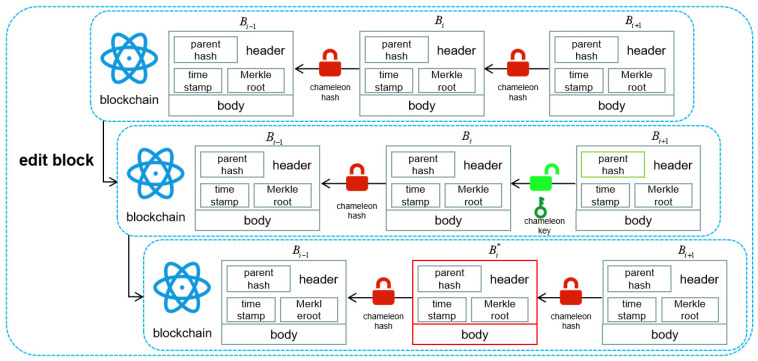
Redactable blockchain editing process.

When the traceability data of agri-food on the first chain exceed the validity period, the data on the chain can be deleted through node proposal. Redaction operations are performed on a redactable blockchain. In the top blockchain, all padlocks are locked, resulting in an immutable blockchain. In the middle blockchain, the padlock from block Bi + 1 to block Bi is open, meaning that the content of block Bi can be redacted. In the bottom blockchain, block Bi is redacted (resulting in block Bi) and all the padlocks are once again locked, making the blockchain immutable, as shown in Figure 4.

The algorithm for redact block of parallel blockchain architecture for agri-food traceability through redactable blockchain based on chameleon hash function is shown in Algorithm 1.
**Algorithm 1:** Chain Redact.**input**: The input overdue block *B*, the redacted block *B′*, and the chameleon hash trapdoor key *tk*. **output**: the calculated redacted block *B′* with salt *sk*. m ← Serialize (B); m′ ← Serialize (B′); h ← Hash (m); h′ ← Hash (m′); b ← j^d mod n; hm ← m—m′; bhm ← b^hm mod n; rm ← tk mod n; sk ← (bhm * rm) mod n; B′ ← ToBlock (B′, sk); B′ ← Deserialization (B′); **return** *B*′

### 4.2. Overdue Data Unloading and Recovery

Due to the traceability characteristics of agri-food, even if the agri-food has expired, it should also be traceable. Therefore, it is necessary to realize the safe recovery of the unloaded data while unloading the expired agri-food traceability data and reducing the storage pressure of the blockchain.

**Figure 5:**
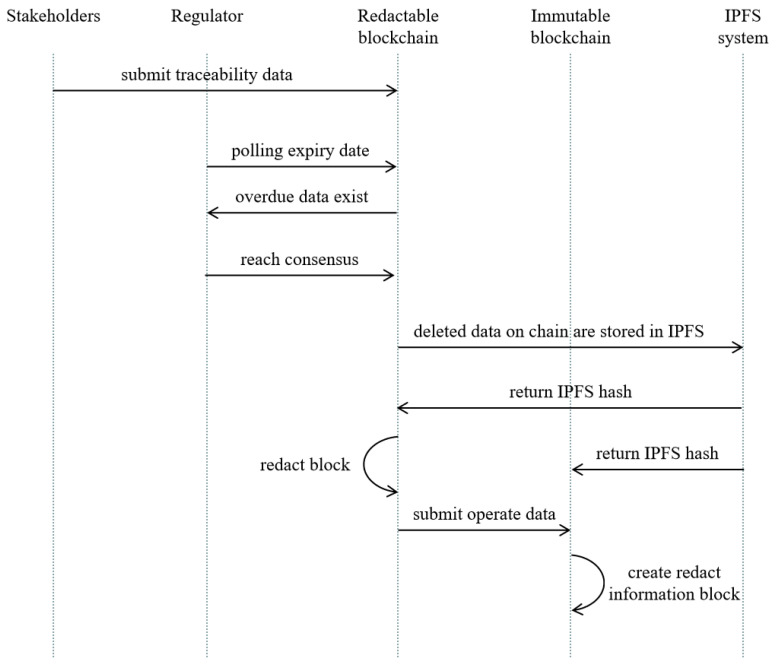
Delete process of expired data.

The flow of the deletion process of expired data is shown in Figure 5.

The main processes are as follows:

- Step 1: Submit agri-food traceable data, namely a traceable data record.
- Step 2: Determine the polling expiry date. Stakeholders regularly poll to check whether there is expired agri-food traceability data on the blockchain.
- Step 3: Judge whether there are expired agri-food traceable data; if so, go to Step 4; otherwise, go to Step 2.
- Step 4: Stakeholders can choose to initiate a request to delete expired data. If the deletion request is made, go to Step 5; otherwise, go to Step 2.
- Step 5: Store the data to be deleted in IPFS to obtain the returned IPFS hash.
- Step 6: Build a new block without expired agri-food traceable data.
- Step 7: Replace old blocks with new blocks without expired agri-food traceable data.
- Step 8: Submit operation data to a non-redactable blockchain.

Algorithms 2 and 3 introduce the concepts of overdue data unloading and accountability for expired data, respectively.
**Algorithm 2:** Overdue data unloading and operation record.**input**: The input overdue block *B*, the redacted block *B′* without salt sk, and the chameleon hash trapdoor key *tk*. **output**: the block of operation record on immutable blockchain *OB*. *B*′ ← chainRedact(*B*, *B′*, *tk*) *hash* ← IPFSUplink(*B*) *OB* ← genBlock() *OB* ← addField (*OB*, *hash, OperatorID*) **return** OB

**Algorithm 3:** Chain Redact.**input**: The IPFS unique identification of information *IPFSHash.* **output**: The recovered block *R.* m ← IPFSQuery(*IPFSHash*); R ← ToBlock(m); R ← Deserialization(R) **return** *R*

### 4.3. Editorial Rights Management

Redactable blockchain is in conflict with the immutability of the blockchain. Therefore, redaction should be carried out under strict constraints. Ciphertext–policy attribute-based encryption (CP-ABE) can achieve fine-grained access control over data. The role of each participant is determined by attributes. Therefore, an access structure is specified to express the authorized attribute set. The set in the access structure is the authorization set; on the contrary, the set not included in the access structure is the non-authorization set. In this encryption algorithm, the access policy is embedded in the ciphertext, and the user’s attributes are embedded in the decryption private key. CP-ABE allows the data encryptor to specify the access structure, and the attributes in the access structure have logical relationships, so this method can meet the fine-grained access control requirements.

CP-ABE includes four basic algorithms: Setup, Encrypt, KeyGen, and Decrypt, as shown in the following formula:

A = (Setup, Encrypt, Key, Decrypt)(6)

The system-setting algorithm takes the security parameter d as the input, whereas the outputs are the encryption public key *Pk* and the master private key *Mk*, which are recorded as follows:

Setup (*d*) → (*Pk*, *Mk*) (7)

Using the public key *Pk*, the chameleon hash key is used as the plaintext information *M*, and the access structure *T* is used to formulate the ciphertext access strategy. The ciphertext output after encryption is *Ct*. Only the supervisor or intermediary involved in the access strategy can decrypt the ciphertext, which is recorded as follows:

Encrypt (*Pk*, *M*, *T*) → *Ct.* (8)

The decryption private key *Sk* can be calculated from the master private key *Mk* and the attribute set *S*. The private key contains the user attribute of the access policy, which is recorded as follows:

KeyGen (*MK*, *S*) → *Sk.* (9)

By accessing the ciphertext *Ct* of structure *T* and decrypting the private key *Sk*, you can judge whether you have permission to access the encrypted data. Without the requisite access permissions, one will be unable to access the encrypted data. If you are authorized, you can decrypt the encrypted data to obtain the chameleon hash info and edit the blockchain, which is recorded as

Decry (*Pk*, *Ct*, *Sk*) → *M* → Info. (10)

**Figure 6:**
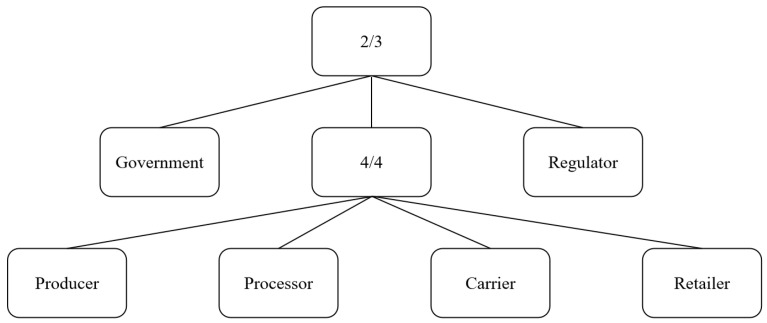
Ciphertext-policy attribute-based encryption access tree.

In general, the expression of access structure can be effectively conveyed using a tree structure. Specifically, the internal nodes of the tree are utilized to represent logical operations such as AND, OR, and threshold operations, while the attributes are represented by the leaf nodes. This tree structure is commonly referred to as an access tree. As depicted in Figure 6, the access tree provides a straightforward example of this structure. The government, the regulator, and all stakeholders were involved in access control. The unloading of expired data can only be completed when both the regulator and the government agree to the modification, or when one of the two agrees to the modification and all stakeholders agree to the modification.

### 4.4. Storage Performance

IPFS is a distributed file system that aims to provide a high-performance and scalable solution for storing and sharing files on the internet. The integration of IPFS and blockchain provides significant advantages in terms of reducing data storage redundancy compared to blockchain alone. In traditional blockchain systems, data redundancy is a critical issue due to the requirement of storing all transaction data on every node. This results in high storage requirements, which can impact network scalability and performance. By utilizing IPFS, data are distributed and stored through a decentralized peer-to-peer network, reducing the need for centralized storage and providing a more efficient and resilient approach to data storage. This approach allows for a reduction in data storage redundancy, as only interested nodes need to store the data, while other nodes can access the data dynamically. Additionally, IPFS provides advanced features such as content addressing, data deduplication, and compression, which further reduces data storage redundancy and improves data transfer efficiency. Moreover, the combination of IPFS and blockchain can improve network scalability, as IPFS can handle large volumes of data and transactions, reducing the burden on the blockchain network. This results in a more efficient and flexible approach to data storage and management, with reduced data storage redundancy and improved network performance.

## 5. Performance Analysis and System Implementation

In this section, the performance of the model is analyzed theoretically and experimentally. The effectiveness of the proposed model is illustrated via comparisons with the state-of-the-art models [26,27,28,29,30].

### 5.1. Experimental Environment and Parameter Settings

To test the performance of the proposed architecture, we built the Ubuntu 16.04 Linux system, used Ethereum as the blockchain platform, and built a redactable Ethereum private chain, an immutable Ethereum private chain, and an IPFS for testing. The Linux nodes we used for testing have four cores in their CPU, eight logical processors, and 16 G of RAM. We also included a solid disk to increase the writing and reading operations. The test data come from the Inner Mongolia Zhaofeng Hetao wheat flour industry tracing platform. The test dataset spans from January 2022 to December 2024, encompassing a total of 36 months of AFSC traceability records, amounting to approximately 360,000 entries. The average storage capacity of a single AFSC traceability record is approximately 350 bytes, and the storage cycle for AFSC traceability information is one year. The test chain has 20 peer nodes. Producers, processors, carriers, and retailers have four peer nodes, respectively. Governments and regulators have two peer nodes, respectively. Each peer node acts as a redactable Ethereum private chain, an immutable Ethereum private chain, and an IPFS node. The specific configuration is shown in Table 2.

### 5.2. Correctness Analysis

Select a pair of permutations (f_0_(*x*), f_1_(*x*)) over a common domain. Make it infeasible to calculate the values *x* and *y* from the domain, such that f(*x*) = f(*y*). (f_0_, f_1_) is the public key and (f_0_^−1^, f_1_^−1^) is the private key. Then, we denote the binary representation of a message m of length k as m = m[1]…m[k] where m[1] is the first message bit and m[k] is the last.

For any pair (m_1_, r_1_), and redactable message m_2_, CHAM-HASH(f_0_, f_1_)(m_1_, r_1_) = CHAM-HASH(f_0_, f_1_)(m_2_, r_2_) can be found; the value r_2_ can be computed as follows:

(11) r2=f(f(…(CHAM−HASH(m1,r1)))m2k′−1−1)m2[k′]−1

Therefore, when the trapdoor key authority is held, the blockchain expired data can be unloaded.

### 5.3. Collision Resistance Analysis

Assume that given (f_0_, f_1_), one can find two pairs of m_1_, r_1_ and m_2_, r_2_ with m_1_ ≠ m_2_, such that CHAM-HASH(f_0_, f_1_)(m_1_, r_1_) = CHAM-HASH(f_0_, f_1_)(m_2_, r_2_). Let *i* be the largest index of a bit where m_1_ and m_2_ differ (i.e., m [*i*] ≠ m [*i*] and m [*j*] = m [*j*] for all *j* > *i*). Such a bit exists due to the suffix-free property. Since we assume that the result of the hash function on (m_1_, r_1_) and (m_2_, r_2_) is the same, and the messages are identical in positions i + 1, …, k, then the result of the computation after the i-th bit must also be the same for both messages. Thus, we found a pair of values r_1_′ and r_2_′, for which f_m1[i]_(r_1_′) = f_m2[i]_(r_2_′), but m_1_[*i*] ≠ m_2_[*i*], in contradiction to the fact that f_0_, f_1_ is a claw-free pair.

Therefore, the traceable data stored in the redactable blockchain cannot be tampered with without the trapdoor key.

### 5.4. Storage Performance Analysis

**Figure 7:**
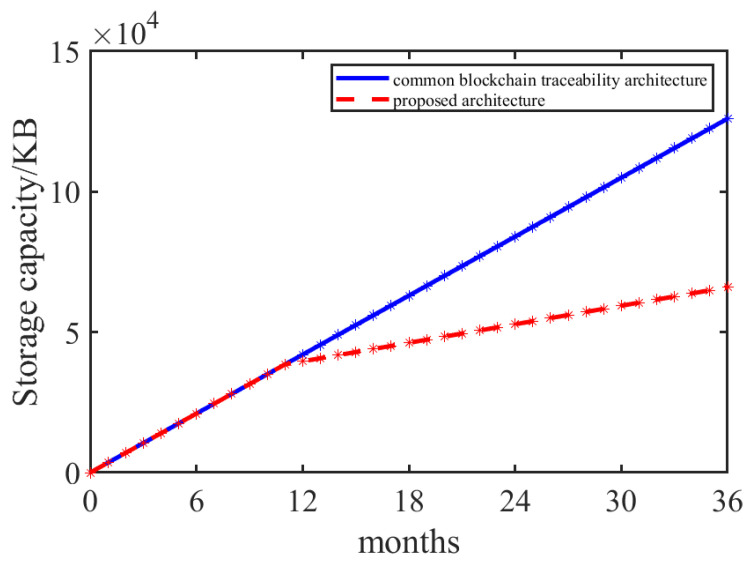
Comparison of storage capacity.

The common formats of agri-food traceability data are shown in Table 3:

Each agri-food traceability datum comprises about 350 bytes. Generally, when the data volume increases steadily, the data storage volume on the blockchain increases linearly over time. In actual production, an average of 10,000 pieces of data are added each month. Figure 7 calculates the comparison between the proposed architecture and the common blockchain traceability architecture in data storage within 36 months. In the first 12 months of system operation, the storage capacity of the proposed architecture is the same as that of the common blockchain traceability architecture because there is no retrospective data beyond the shelf life. Starting from the 13th month, due to the unloading of expired data to IPFS, the storage capacity of the proposed architecture is lower than that of the traditional blockchain traceability architecture. When the 36th month of system operation, the storage size of the traditional blockchain traceability architecture reaches 123,156.21 KB, while the storage size of the proposed architecture is 64,509.82 KB. The storage capacity of the proposed architecture is 52.38% of that of the traditional blockchain traceability architecture.

### 5.5. Retention Time Performance Analysis

**Figure 8:**
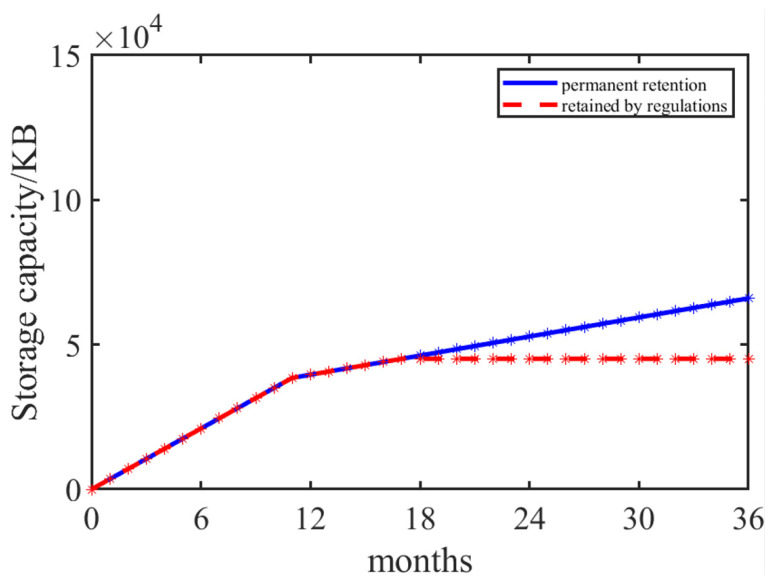
Comparison of retention method.

Countries have certain requirements for the storage time of agri-food traceability data, and it is not necessary to permanently store agri-food traceability data. For example, according to the Food Safety Law of the People’s Republic of China, agri-food traceability data should be kept for at least 6 months after the expiration of the warranty period. If there is no clear warranty period, the agri-food traceability data should be kept for 18 months. The European food safety law requires that records of traceability data for agri-food be kept for at least five years. Therefore, companies may choose to delete traceability data of agri-food after a retention period that exceeds the legal requirements. Of course, companies may also set higher corporate standards based on legal requirements, such as permanently retaining traceability data. Figure 8 calculates the comparison of data storage capacity when the traceability data of agri-food are only saved for up to 6 months after the expiration date and when the traceability data of agri-food are permanently retained for permanent accountability. It can be observed that during the first 18 months of system operation, the storage capacity for permanent retention is equal to that specified by the regulations pertaining to retainment, as there were no retrospective data that could be deleted in accordance with legal requirements. Beginning in the 19th month, the storage capacity regulations became constant, while the storage capacity of permanent retention was found to be linear.

### 5.6. Query Efficiency Analysis

**Figure 9:**
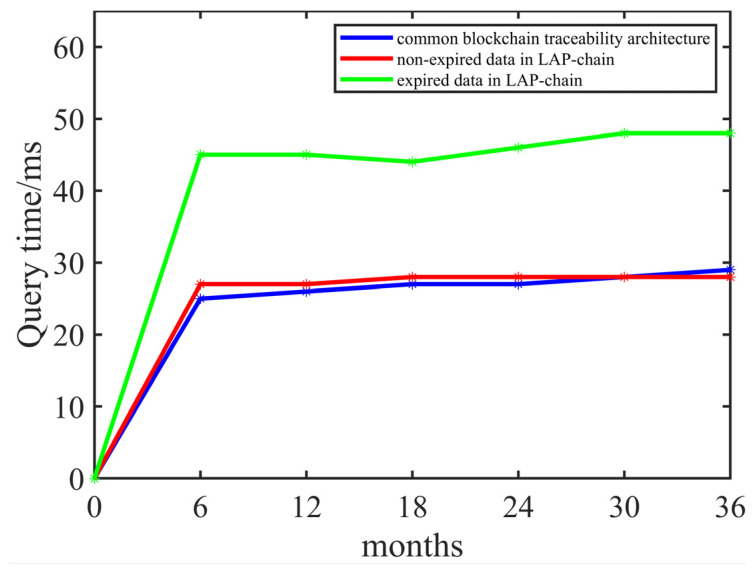
Comparison of query efficiency.

Figure 9 compares the proposed architecture with the common blockchain traceability architecture in query efficiency.

The experimental results indicate that in the early stages of system operation, the query efficiency of non-expired data in LAP-chain is slightly lower than that of the common blockchain traceability architecture. However, as the system operates, the data storage volume in LAP-chain becomes lower than that of the common blockchain traceability architecture, leading to LAP-chain surpassing the common blockchain traceability architecture in query efficiency. Additionally, the query time for expired data in LAP-chain is significantly higher than in the common blockchain traceability architecture. However, in practical applications, the query frequency for expired data is extremely low, ensuring that LAP-chain meets the requirements of real-world usage.

### 5.7. Advantages Compared with Other Architectures

Table 4 compares the schemes proposed in this paper with those proposed in other papers in terms of storage, consumption, efficiency, compatibility, effectiveness, and recoverability.

### 5.8. System Implementation

**Figure 10:**
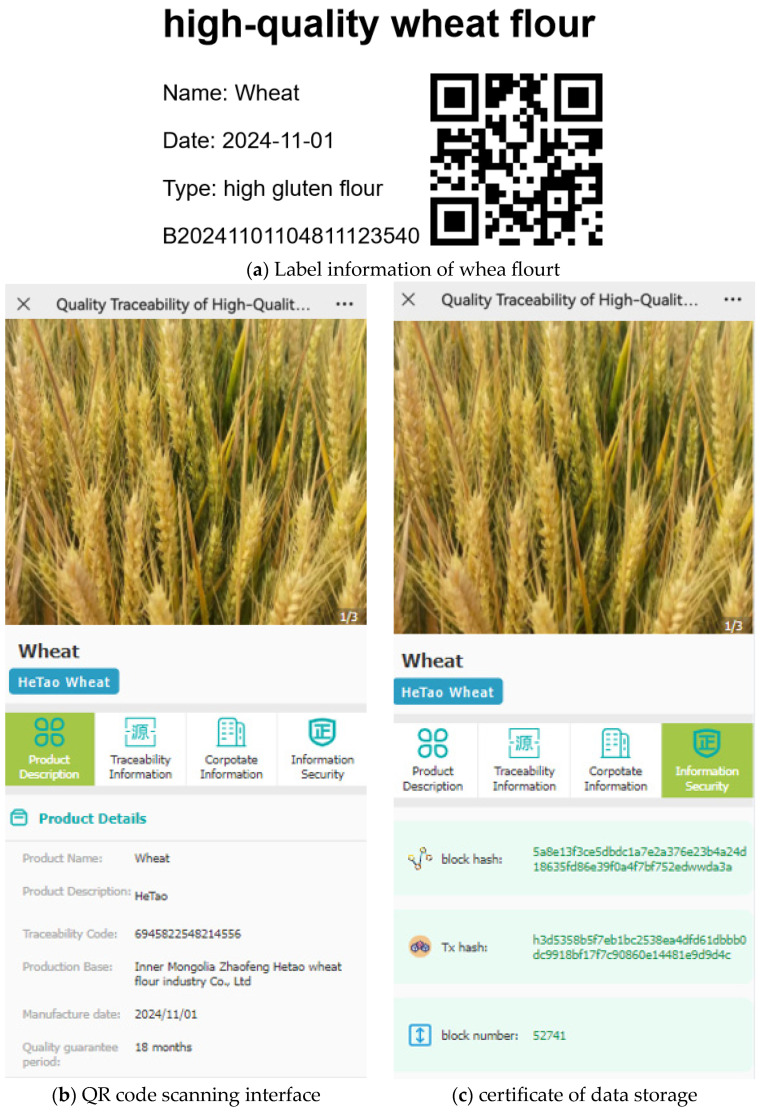
Wheat blockchain traceability system.

The system has been successfully applied to Hetao Wheat in Zhaofeng City, Inner Mongolia Province, China. The system is designed to collect information on the supply chain of the wheat product, from planting and warehousing to processing, transportation, and sales, using the LAP-chain method. the LAP-chain reduces the storage pressure of blockchain equipment and ensures data accountability. A wheat flour traceability label and related traceability information are presented in Figure 10. The label information of wheat, as depicted in Figure 10a, contains a QR code that enables users to send a query request to the background server and obtain relevant traceability information. Figure 10b presents a schematic diagram of the mobile-based QR code scanning interface, while Figure 10c offers information on the product stored on the blockchain and the certificate of data storage.

## 6. Conclusions

In conclusion, this paper presents a comprehensive analysis of the key links in agri-food traceability and proposes a solution to improve the timeliness and accountability of traceability data by using blockchain technology. Specifically, we propose LAP-chain, a lightweight accountable parallel blockchain architecture, which reduces the storage pressure of the blockchain equipment while ensuring the accountability of data by offloading expired data to the InterPlanetary file system. Our experimental results show that the LAP-chain built on the Ethereum private chain has good correctness, collision resistance, and storage performance. Our proposed solution has the potential to enhance the efficiency and transparency of agri-food traceability systems and can contribute to improving the safety and quality of agri-food.

However, this study has certain limitations. First, the current implementation focuses on a single commodity scenario (e.g., wheat), and its applicability to more complex, multi-commodity supply chains remains to be validated. This is particularly true in the context of international agri-food trade, addressing the unique agri-food challenges faced by different regions. Second, while IPFS addresses storage scalability, long-term data availability and retrieval latency in decentralized storage systems require further investigation. Third, the economic feasibility of deploying LAP-chain in real-world agri-food supply chains, particularly for small-scale farmers, needs to be evaluated. Lastly, while redactable blockchain enables authorized data modification, it also introduces the risk of malicious actors exploiting editorial rights. Although this approach proposes a CP-ABE-based key management scheme, it is not applicable to all agri-food traceability architectures. Therefore, there is a need for further research on the management scheme for editing rights in a general architecture.

Future research directions include extending LAP-chain to support multi-commodity traceability, optimizing IPFS integration for improved data retrieval performance, and conducting cost–benefit analyses to assess the economic viability of large-scale deployment. Additionally, exploring the integration of emerging technologies, such as edge computing and artificial intelligence, could further enhance the scalability and intelligence of agri-food traceability systems. By addressing these challenges, LAP-chain has the potential to significantly contribute to improving the safety, quality, and sustainability of global agri-food supply chains.

## Figures and Tables

**Table 1:** Data lifetime of key links.

Key Link		Definite Lifetime	Indefinite Lifetime
Producers		Seed source, wheat variety, planting time, harvest time, planting operation, fertilizer information and use records, pesticide information and use records, operators, and product batch number.	Basic information of producer, planting method, contact information of producer, planting base name, planting base address, and base soil quality.
Processors	Wheat wetting	Wheat wetting method (dry and wet), water temperature, times of watering, duration of wheat wetting, and wheat wetting machine information.	Basic information, responsible person, contact information, and license information of the processor.
	Milling	Milling method (simplified flour grading, medium flour grading, strengthened flour grading, and balanced powder discharging by grinding collision), number of passes of skin grinding system, contact length of grinding roller, and machine information.	
	plansifter	sieve size, physical and chemical indexes of flour, and plansifter machine information.	
	Packing	Commodity name, processing personnel information, manufacturing date, shelf life, expiry date, and product batch number.	
Carriers		Transport vehicle, logistics number, car number, place of departure, destination, the person in charge, and product batch number.	Name of logistics enterprise, address of logistics company, license information, and contact information.
Retailers		Product name, product quantity, purchase time, product storage time, product storage location, delivery time, and product batch number.	merchant name, shop address, store principal information, business license information, and merchant contact information.

**Table 2:** Parameter configuration.

Parameter	Value
Linux version	16.04
Geth version	1.10.17
Go version	1.17.7
Consensus mechanism	PoA
Peer number	20
Period	30
Epoch	30,000

**Table 3:** Traceability data details on the blockchain.

Traceability Data Details on the Blockchain
productBatchNum:B20241101104811123540|productionNum:202403031245215|wheatVariety:JM22|plantingTime:20240601|harvestTime:20240801|processingNum:2024110102152412|wheatWettingMethod:wet|millingMethod:simplifiedFlourGrading|plansifterSieveSize:1|manufacturingDate:20241101|shelfLife:6|expiryDate:20250501|logisticsNumber:L202411011542581|carNumber:J51478|productName:wheat

**Table 4:** Comparison of important related work.

	[26]	[27]	[28]	[29]	[30]	Our
Storage	High	Middle	**Low**	**Low**	**Low**	**Low**
Consumption	High	High	High	High	**Low**	**Low**
Efficiency	High	High	High	High	**Low**	**Low**
Compatibility	**Yes**	No	No	No	**Yes**	**Yes**
Effectiveness	**Yes**	**Yes**	**Yes**	**Yes**	**Yes**	**Yes**
Recoverability	**Yes**	**Yes**	No	No	No	**Yes**

## Data Availability

The original contributions presented in the study are included in the article, further inquiries can be directed to the corresponding author.
